# Cardiovascular Events in Patients with Severe Asthma—A Retrospective Study of Two Cohorts: Asthma Type T2 Treated with Biologics and Non-Type T2

**DOI:** 10.3390/jcm13154299

**Published:** 2024-07-23

**Authors:** Paula Granda, Elena Villamañán, Daniel Laorden, Carlos Carpio, Victoria Collada, Javier Domínguez-Ortega, Leticia de las Vecillas, David Romero-Ribate, Omar Fabián Chaparro-Díaz, Teresa Lázaro Miguel-Sin, Daniela Jose Alloca-Álvarez, Jorge Mauricio Correa-Borit, Itsaso Losantos, Patricia Mir-Ihara, Emilio José Narváez-Fernández, Santiago Quirce, Rodolfo Álvarez-Sala

**Affiliations:** 1 Pharmacy Department, Gómez Ulla Military Hospital, 28047 Madrid, Spain; 2 Pharmacy Department, La Paz University Hospital, IdiPAZ, 28029 Madrid, Spain; elena.villamanan@salud.madrid.org (E.V.); victorialucia.collada@salud.madrid.org (V.C.); 3 Medicine Department, Autonomous University of Madrid, 28049 Madrid, Spain; carlinjavier@hotmail.com (C.C.); rodolfo.alvarezsala@salud.madrid.org (R.Á.-S.); 4 Pneumology Department, La Paz University Hospital, IdiPAZ, 28046 Madrid, Spain; laordendaniel@gmail.com (D.L.); davizrom@hotmail.com (D.R.-R.); fabianchaparrodiaz@gmail.com (O.F.C.-D.); telazmig95@gmail.com (T.L.M.-S.); alloccadj@gmail.com (D.J.A.-Á.); 5 Allergy Department, La Paz University Hospital, IdiPAZ, 28046 Madrid, Spain; javier.dominguez@salud.madrid.org (J.D.-O.); leticia.delasveci@gmail.com (L.d.l.V.); jorge_correa_borit@hotmail.com (J.M.C.-B.); patriciakmir@gmail.com (P.M.-I.); emilionarvaez2015@hotmail.com (E.J.N.-F.); squirce@gmail.com (S.Q.); 6 Biostatistics Department, La Paz University Hospital, 28046 Madrid, Spain; itsaso.losantos@salud.madrid.org

**Keywords:** asthma, cardiovascular disease, T2 asthma

## Abstract

**Background**: The prevalence of cardiovascular events (CVEs) in patients with asthma varies amongst studies, with little evidence as to their prevalence in patients treated with monoclonal antibodies (mAbs). In this retrospective, observational study, we aimed to evaluate the prevalence of CVEs in patients with T2 and non-T2 asthma and to identify risk factors associated with CVEs. **Methods**: A total of 206 patients with severe asthma were included. Demographic variables, respiratory comorbidities and cardiovascular risk factors were collected, along with respiratory function, laboratory parameters and respiratory pharmacotherapy, including treatment with mAbs. **Results**: A total of 10.7% of the patients had any CVE from the date of asthma diagnosis, with a higher risk in those patients with chronic obstructive pulmonary disease (odds ratio [OR] = 5.36, 95% CI 1.76–16.31; *p* = 0.003), arterial hypertension (OR = 2.71, 95% CI 1.13–6.55; *p* = 0.026) and dyslipidaemia (OR = 9.34, 95% CI 3.57–24.44; *p* < 0.001). No association between mAb treatment and a CVE or between time of mAb treatment and the event was found. No significant differences were observed between the T2 and non-T2 cohort. After a multivariate analysis, dyslipidaemia was identified as an independent risk factor (OR = 13.33, 95% CI 4.49–39.58; *p* < 0.001), whereas regular use of inhaled corticosteroids was associated with a reduced risk of a CVE (OR = 0.103, 95% CI 0.021–0.499; *p* = 0.005). Further research is needed to fully understand the relationship between severe asthma and CVEs. **Conclusions**: This study suggests that patients with severe asthma experience a higher percentage of CVEs compared with the general population.

## 1. Introduction

Non-respiratory comorbid conditions in asthma include cardiovascular disease [1,2]. Several studies [3,4,5,6,7,8,9,10,11] have shown an association between asthma and cardiovascular events (CVEs) and, more specifically, that this association is particularly observed in women with adult-onset asthma [2]. The T2 asthma phenotype (represented by allergic and eosinophilic asthma) is considered a risk factor for coronary artery disease; in particular, uncontrolled eosinophilic asthma has been related to the development of coronary artery obstruction and vasospasm without obstruction [12,13,14]. However, CVE prevalence in patients with asthma varies between studies, and robust evidence of this relationship is limited [15].

Moreover, there is little evidence of the risk of a CVE in patients with severe asthma treated with monoclonal antibodies (mAbs); thus, the association between a CVE and these drugs remains unknown. Restrictive inclusion and exclusion criteria are common factors that prevent the extrapolation of randomised controlled trial (RCT) results to normal clinical practice. Given that patients with severe asthma typically present several comorbidities [15], many of them are often not eligible for these RCTs [16,17,18]; thus, the results are not fully representative of the unselected real-world population.

## 2. Aim

Given the scarce evidence in this regard, in this retrospective, real-life study, we aimed to evaluate the prevalence of CVEs in patients with severe asthma and to identify the risk factors associated with CVEs in this population.

## 3. Materials and Methods

### 3.1. Study Design

We performed a retrospective, cross-sectional study involving patients diagnosed with T2 and non-T2 severe asthma in the multidisciplinary severe asthma unit of a Spanish tertiary care hospital. 

### 3.2. Study Participants

The inclusion criteria were patients aged older than 18 years and with a diagnosis of severe asthma according to the European Respiratory Society/American Thoracic Society guidelines [19]. The exclusion criteria were incomplete medical history, as well as patients with palliative comorbidities such as lung cancer, chronic obstructive pulmonary disease (COPD), cystic fibrosis or other systemic palliative diseases. This study was conducted under standard clinical practice conditions, without any additional procedures or interventions in daily practice.

### 3.3. Clinical Outcomes and Analysis

In accordance with the clinical guideline recommendations in force at the time of the study [20,21], the patients diagnosed with T2 severe asthma were grouped into a T2 cohort, all of whom were receiving biologic therapy (omalizumab, reslizumab, mepolizumab or benralizumab); the patients diagnosed with non-T2 severe asthma, who were not candidates for biologic therapy, formed a non-T2 cohort.

Patients diagnosed with non-T2 severe asthma receiving biologic therapy, those with a T2 profile not receiving biological treatment and those diagnosed with bronchial asthma receiving biological treatment for another pathology were excluded from the analysis. Patients with a prior history of a CVE before asthma diagnosis were also excluded. 

The demographic and clinical variables collected included time from asthma onset, sex, respiratory comorbidities (COPD, allergic asthma, obstructive sleep apnoea [OSA] syndrome, rhinosinusitis and nasal polyposis) and cardiovascular risk factors (smoking status, hypertension, dyslipidaemia, diabetes mellitus and obesity). Respiratory function variables were measured: forced expiratory volume in the first second (FEV1), forced vital capacity (FVC) and the FEV1/FVC ratio. Blood eosinophil count, immunoglobulin E levels and fractional exhaled nitric oxide levels were also collected.

CVEs were defined as angina with coronary revascularisation, myocardial infarction, heart failure requiring hospitalisation, supraventricular arrhythmia, pulmonary embolism or stroke. Data were obtained from electronic medical records, the patient’s treatment history registry and electronic pharmacy refill data.

To account for differences amongst subgroups with a similar CVE risk, we stratified both cohorts by age (18–44 years and ≥45 years) and sex.

### 3.4. Statistical Analysis

The normality of the data distribution was assessed with the Kolmogorov–Smirnov test. Parametric variables were expressed as means (standard deviation [SD]), and nonparametric variables were expressed as medians (interquartile ranges [IQRs]).

Categorical variables were expressed as frequencies and percentages and analysed by the chi-squared test or Fisher’s exact test when appropriate. We calculated 95% confidence intervals when appropriate. Differences were considered significant at a *p*-value < 0.05 (two sided).

We conducted a multivariable analysis using age, allergic asthma, arterial hypertension, dyslipidaemia, COPD and inhaled corticosteroid use as independent variables, and a CVE as the dependent variable.

A multivariable analysis that used age, arterial hypertension, dyslipidaemia, COPD, OSA and tobacco use as independent variables and a CVE as the dependent variable was also conducted in the T2 cohort.

The purpose of these multivariable analyses was to determine which clinical variables in patients with severe asthma might be related to a CVE.

All data were analysed using the SAS 9.1 program (SAS Institute Inc., Cary, NC, USA).

## 4. Results

### 4.1. Patient Demographics and Clinical Characteristics

A total of 206 patients with severe asthma were included; 135 (65.6%) were female. The median age of the patients was 59 (IQR 48–70) years. Clinical characteristics and comorbidities are shown in Table 1.

### 4.2. Cardiovascular Events

The primary outcome was the proportion of patients with severe asthma who developed any CVE from asthma diagnosis to the present based on the inflammatory phenotype: T2 versus non-T2. A total of 22 (10.6%) patients had a CVE; CVE distribution by age and sex according to the inflammatory phenotype is shown in Table 2, where we find a higher prevalence in the group of women aged ≥45 years. 

In the T2 cohort, we found a greater number of patients who had experienced a CVE in contrast with the non-T2 cohort; however, it was not significant (*p* = 0.399, Table 3). 

Considering the whole cohort, in terms of comorbidities (Table 3), a higher statistically significant risk of presenting a CVE was observed in those patients with COPD (odds ratio [OR] = 5.36, 95% CI 1.76–16.31; *p* = 0.003). Arterial hypertension (OR = 2.71, 95% CI 1.13–6.55; *p* = 0.026) and dyslipidaemia (OR = 9.34, 95% CI 3.57–24.44; *p* < 0.001) were statistically associated with CVEs. No other significant association was found between comorbidities or the cardiovascular risk factors analysed and CVEs in patients with severe asthma.

In contrast, a reduced risk of CVEs was observed in those patients who had been treated with inhaled corticosteroids (OR = 0.19, 95% CI 0.05–0.71; *p* = 0.007).

Considering the T2 cohort, in patients with severe asthma treated with mAbs, a respiratory comorbidity such as COPD (OR = 8.90, 95% CI 2.42–32.70; *p* = 0.001) was statistically associated with a higher CVE risk. We also found this higher risk in patients with arterial hypertension (OR = 5.39, 95% CI 1.83–15.92; *p* = 0.002) or dyslipidaemia (OR = 15, 95% CI 4.46–50.48; *p* < 0.001).

We did not find any association between biologic treatment and CVEs (*p* = 0.206). Also, we found by Cox regression that there was no association between the time of treatment with a biologic and the CVE (*p* = 0.143).

### 4.3. Multivariate Logistic Regression Analysis

Dyslipidaemia was identified as a risk factor associated with CVEs (Table 4), whereas the use of inhaled corticosteroids protects against them.

Patients with dyslipidaemia were at an increased risk of a CVE in the T2 cohort, in which we also found age to be a risk factor.

The confidence intervals were wide for dyslipidaemia, which was reflective of the relatively small sample size.

## 5. Discussion

Several previous studies have observed a positive association between asthma and the incidence of any CVE [22]. Amongst the plausible mechanisms suggested is the chronic airway inflammation observed in respiratory tract diseases, which could contribute to systemic inflammation, increasing arterial stiffness, an established risk factor for CVEs that increases with disease severity [23,24].

As expected, our findings support the hypothesis of a higher CVE risk in patients with severe asthma, given that 10.7% of them had any CVE from the date of asthma diagnosis, a higher percentage than that observed in the general European adult population [25]. Although the pathogenetic mechanisms underlying this association are not fully understood, they are believed to be related to the shared inflammatory pathways in both conditions [15].

Hyperplasia and the abnormal contraction of smooth muscle cells, resulting in airway constriction in asthma, are characteristics shared with the vascular remodelling and randomised irregularities observed in cardiovascular diseases [15]. Uncontrolled asthma, especially in patients exhibiting a T2 inflammatory profile or marked eosinophilic activation, has the potential to induce a procoagulant state [2,15]. Indeed, we found a higher number of patients who had experienced a CVE in the T2 cohort in contrast with the non-T2 cohort; however, it was not significant, probably because of the low number of cardiovascular events recorded.

Kounis syndrome, a hypersensitivity coronary disorder, is characterised by the co-occurrence of an acute coronary syndrome and an allergic reaction [26]. It has three main variants: type I, which involves coronary spasm without pre-existing coronary disease; type II, which occurs in the presence of atheromatous disease; and type III, which is associated with drug-eluting stent thrombosis [27]. The pathophysiology of the syndrome involves mast cells, platelets and eosinophils [28]. Although Kounis syndrome is typically associated with acute coronary syndrome and anaphylactic shock, it can also be present in cardiogenic shock [29]. This syndrome might also explain the higher incidence of cardiovascular disease (CVD) in patients with T2 asthma, as it is mediated by an inflammatory mechanism [26].

Iribarren et al. [30] found a statistically significant association between asthma and coronary heart disease in women, which is consistent with previous studies [30,31,32,33,34,35]. Our results showed a higher risk of a CVE in women (15/22, 68.2%), mainly with T2 asthma (11/15, 73.3%), although this association was not statistically significant.

Although no significant differences were observed between the T2 and non-T2 cohorts, we characterised the CVE risk of patients with T2, focusing on previous studies [2,15]. However, the CVE risk in patients with severe asthma treated with mAbs had not yet been studied. Biologic treatments target specific inflammatory pathways involved in asthma, particularly in patients with T2 inflammation [36]; this could lead to a reduction in inflammation and therefore CVD. However, the relationship between biologic treatments and CVD in patients with asthma is complex, with shared pathogenic pathways between asthma and CVEs [15]. An observational study performed to assess the long-term safety and effectiveness of omalizumab in a clinical practice setting demonstrated a higher incidence of CVEs in patients treated with omalizumab [36]. However, confounding factors between patients treated and not treated with omalizumab were likely to explain those results, as Quinta et al. [37] found in their real-world study. They also reported that CVEs were not more likely to be found with anti-IL-5/IL-5Ra therapies compared with omalizumab, except for ischaemic stroke. However, we did not find any association between biologic treatment and CVEs (*p* = 0.206). Also, we found no association by Cox regression between the time of treatment with a biologic and the CVE (*p* = 0.143). 

In addition to the mentioned prothrombogenic mechanisms and inflammatory status, dyslipidaemia has also been suggested to explain the increased risk of CVD among individuals with asthma [22]. A multivariate analysis was performed, selecting factors in the univariate analyses associated with CVEs (*p* < 0.05) and identifying dyslipidaemia as an independent risk factor. Some univariately associated variables were not significant in the multivariate model, likely due to the overlapping nature of cardiovascular comorbidities. Identifying these risk factors could be important for preventing CVEs in patients with asthma.

Given that this was a cross-sectional study, causal relationships cannot be presumed; nevertheless, the observed relationships are likely to be a consequence of severe asthma. In the T2 cohort, age was also identified as an independent risk factor, which is not surprising because it is well known that age is associated with an increased cardiovascular risk [38].

We studied survival by Cox regression and did not find significant results, probably due to the small number of cases. In agreement with other authors [39], we found that regular use of inhaled corticosteroids was associated with a reduced risk of CVEs. This positive effect could be explained by possible anti-inflammatory effects. Suissa et al. [39] observed that in patients using inhaled corticosteroids, the rate of myocardial infarction decreased by 12% for every additional canister of inhaled corticosteroids used during the year. However, the use of short-acting ß(2)-adrenergic agonists, which are commonly used in asthma treatment, has been associated with an increased risk of cardiovascular events [40], although we did not find any associations in our study.

Limitations: The small size of the study population is a limitation of this study and should be taken into consideration when interpreting the results, as should the retrospective design of this study. Further research with a larger sample and prospective design is needed to fully understand the relationship between severe asthma and CVD, highlighting the convenience of including patients treated with biologics to develop appropriate management strategies.

## 6. Conclusions

This study suggests that patients with severe asthma experience a higher percentage of CVEs compared with the general population. Significant risk factors include COPD, hypertension and dyslipidaemia, whereas inhaled corticosteroids are associated with a reduced risk of CVEs. Although more CVEs were observed in the T2 asthma cohort, no significant association with biologic treatments was found. Further studies are needed to fully understand the relationship between severe asthma and cardiovascular disease.

## Figures and Tables

**Table 1:** Patients’ clinical characteristics.

Clinical Characteristic	All Patients with Severe Asthma *n* = 206
Age, years, median (IQR)	59 (48 to 70)
Female, *n* (%)	135 (65.6)
Biologic treatment, *n* (%)	
Omalizumab	111 (53.9)
Mepolizumab	28 (13.6)
Benralizumab	15 (7.3)
Reslizumab	4 (1.9)
Comorbidities, *n* (%)	
Allergic asthma	121 (58.7)
Obesity/overweight	98 (47.6)
Diabetes	24 (9.2)
Hypertension	65 (31.6)
Dyslipidaemia	52 (25.2)
OSA	21 (10.2)

Abbreviations: OSA, obstructive sleep apnoea syndrome.

**Table 2:** Cardiovascular events (CVEs) by age and sex.

CVEs	Total		T2 Severe Asthma		Non-T2 Severe Asthma	
	18–44 yrs *n* = 38	>45 yrs *n* = 168	18–44 yrs *n* = 29	>45 yrs *n* = 135	18–44 yrs *n* = 9	>45 yrs *n* = 33
All patients *n* (95% CI)	1 (2.7%, 95% CI 4.8–13.8)	21 (12.8%, 95% CI 8.5–18.8)	0 (0%, 95% CI 0–12.1)	16 (12.1%, 95% CI 7.6–18.8)	1 (11.1%, 95% CI 1.9–43.5)	5 (15.6%, 95% CI 6.9–31.7%)
Women	0 (0%, 95% CI 0–14.8)	15 (13.5%, 95% CI 8.4–21.1)	0 (0%, 95% CI 0–17.6)	11 (12.6%, 95% CI 7.2–21.2)	0 (0%, 95% CI 0–48.9)	4 (16.7%, 95% CI 6.7–35.9)
Men	1 (6.7%, 95% CI 1.2–29.8)	6 (11.3%, 95% CI 5.3–22.6)	0 (0%, 95% CI 0–27.7)	5 (11.1%, 95% CI 4.8–23.5)	1 (20%, 95% CI 3.6–62.5)	1 (12.5%, 95% CI 2.2–47.1)

**Table 3:** Univariate odds ratios for cardiovascular events amongst patients with asthma.

Total (*n* = 206)				T2 Severe Asthma (*n* = 164)		
*Characteristic*	OR	95% CI	*p* Value	OR	95% CI	*p* Value
Respiratory comorbidities						
Age	1.044	1.013–1.077	0.006	1.094	1.042–1.148	<0.001
Sex			0.715			0.782
T2 asthma			0.399			
Eosinophilic asthma			0.169			0.616
Allergic asthma			0.089			0.169
OSA			0.177			0.061
COPD	5.358	1.761–16.305	0.003	8.896	2.420–32.697	0.001
Nasal polyps			0.944			0.751
Rhinosinusitis			0.620			0.722
Cardiovascular risk factors						
Obesity (BMI > 30)			0.228			0.354
Tobacco use			0.275			0.097
Type II diabetes			0.255			0.276
Arterial hypertension	2.717	1.126–6.551	0.026	5.392	1.826–15.922	0.002
Dyslipidaemia	9.339	3.569–24.439	<0.001	15.95	4.457–50.478	<0.001
Laboratory parameters						
Eosinophils			0.466			0.816
Immunoglobulin E			0.204			0.281
Respiratory functional and asthma control parameters						
Exacerbations			0.936			0.469
FEV1			0.794			0.831
FeNO			0.162			0.299
Respiratory pharmacotherapy						
Inhaled corticosteroids	0.187	0.050–0.705	0.007			0.999
LABA			0.170			0.139
Oral corticosteroids			0.120			0.278
Montelukast			0.421			0.511
SABA			0.170			1

Abbreviations: OSA, obstructive sleep apnoea syndrome; COPD, chronic obstructive pulmonary disease; FEV1, forced vital capacity; FeNO, fractional exhaled nitric oxide; LABA, long-acting beta-agonists; SABA, short-acting ß(2)-adrenergic agonists.

**Table 4:** Results from multivariate logistic regression model.

Total (*n* = 206)				T2 Severe Asthma (*n* = 164)			
Variable	OR	95% CI	*p* Value	Variable	OR	95% CI	*p* Value
Dyslipidaemia	13.328	4.488–39.580	<0.001	Dyslipidaemia	9.885	2.711–36.040	0.001
Inhaled corticosteroids	0.103	0.021–0.499	0.005	Age	1.099	1.033–1.168	0.003

## Data Availability

The data presented in this study are available on request from the corresponding author. The data are not publicly available due to privacy.
